# Taxonomic diversity in the global wheat phyllosphere mycobiome – a meta analysis

**DOI:** 10.3389/fpls.2025.1597807

**Published:** 2025-07-30

**Authors:** Marie Højmark Fischer, Agnieszka Rzepczynska, Rasmus Kjøller

**Affiliations:** ^1^ Section of Terrestrial Ecology, Institute of Science, Department of Biology, Copenhagen University, Copenhagen, Denmark; ^2^ Section of Microbial Ecology, Department of Biology, Lund University, Lund, Sweden

**Keywords:** wheat, phyllosphere, fungi, mycobiome, datamining, meta-analysis

## Abstract

Wheat (*Triticum aestivum L.*) is a major crop grown on all continents. Due to environmental concerns, it is desirable to reduce the inputs of both chemical pesticides and inorganic fertilizers. However, yield reduction must be expected when switching to low-input systems. To mitigate such losses, the use of natural or introduced microbiomes may provide the key to maintaining sustainable yield. Phyllosphere fungi, both endophytic and phylloplane-associated, colonize aboveground plant structures, some of which have the potential to mitigate biotic and abiotic stressors. A first step toward realizing the potential of the wheat microbiome is to map the current knowledge on wheat phyllosphere fungi. This meta-analysis aims to map the diversity and abundance of fungal taxa associated with the wheat phyllosphere across global wheat-producing areas. To this end, we searched previous published literature and retrieved fungal community data from relevant studies. Retrieved studies included both culturing-based and metabarcoding amplicon sequence-based studies. We retrieved and analyzed 33 studies from five regions across the world, which differed greatly in their taxonomic composition. Across all regions, we found that while the majority of identified genera were unique to individual studies, some genera occurred across all five wheat growing regions, specifically *Alternaria, Aspergillus, Bipolaris, Candida, Chaetomium, Cladosporium, Epicoccum, Fusarium, Nigrospora, Penicillium, Pyrenophora, Stemphylium* and *Trichoderma.* Furthermore, we identified that while community composition differed between wheat growing regions, the identification method used was the most significant factor determining the depiction of community composition. We also highlight a lack of research in important wheat growing regions that are important for global wheat production. These considerations and other knowledge gaps are used to pinpoint future research.

## 1 Introduction

Wheat (*Triticum aestivum L*.) is one of the most widely cultivated crops, providing a stable source of nutrients for approximately 40 percent of the worlds population (Acevedo et al., 2018). With more than 218 million hectares across multiple climatic regions, it covers more land than any other commercial crop (Giraldo et al., 2019). Since the green revolution in the 1960s, global wheat production has increased tremendously to approximately 790 million metric tons annually (USDA, 2024), largely due to the increased input of mineral fertilizers and synthetic pesticides (Acevedo et al., 2018). Owing to environmental concerns, reduced inputs of both chemical pesticides and inorganic fertilizers are needed but, 18% percent of the annual wheat production is currently lost to fungal diseases (Savary et al., 2019) and without pesticides, current yield losses are expected to increase significantly (European Commission et al., 2022; Henning et al., 2021). Thus, alternative strategies are needed.

One promising approach is the utilization of plant-associated microbiomes, particularly the fungal communities inhabiting the wheat phyllosphere. The phyllosphere encompasses all aboveground plant structures, including leaves, stems, and flowers, and hosts diverse microbial populations. These microbial populations include fungi and bacteria, both residing on the surface (phylloplane) and within leaf tissues (endosphere) (Sohrabi et al., 2023). These fungi exhibit varied ecological roles, including mutualism, saprotrophy, and pathogenicity. Many endophytic fungi, for example, have been shown to benefit their hosts in various ways (Peñuelas and Terradas, 2014). These benefits include host growth promotion, increased stress resistance, and protection against fungal diseases and insect damage (Hawkes et al., 2021). Pathogen infection resistance can be induced directly, for example, through secondary metabolites released by the fungal endosymbiont, and indirectly by inducing physiological changes in the plant host (Pusztahelyi et al., 2015). The presence of endophytic fungi also battles pathogens through ecological mechanisms, by occupying the niche in the plant host, thus preventing other pathogens from establishing (Gao et al., 2010). Understanding the diversity and distribution of wheat-associated fungi is crucial for harnessing their potential in sustainable agriculture.

Historically, studies of the wheat phyllosphere mycobiome relied on culturing techniques, favoring culturable fast-growing and high-nutrient-favoring organisms (Rhoads et al., 2012). In recent years, meta-amplicon and meta-genome sequencing have allowed for taxonomic identification of whole communities without this bias albeit primer biases and sequencing errors remains an issue (Forry et al., 2024). Determining whether a microbe colonizes the phylloplane, endosphere, or both, is also a challenge. Most studies either identify all microbes in the phyllosphere or attempt to only address endophytes by surface sterilization of the plant material used before isolation or amplification.

Despite growing interest in the wheat phyllosphere microbiome on a local level, the taxonomic composition and geographic variation on a global level remain poorly characterized. A comprehensive synthesis of available data is necessary to identify patterns, research gaps, and future directions. In this review, we present the current knowledge based on the taxonomic composition of the wheat mycobiome identified from previous studies. We mined through publicly available, peer-reviewed literature on the above-ground mycobiome of wheat. We included studies in which fungi were isolated into pure cultures, followed by morphological identification or identification following Sanger sequencing of the ITS region, and more recent studies that implemented meta-barcoding identification methods. Specifically, we asked the following questions: 1) How is research on the wheat phyllosphere mycobiome distributed geographically? 2) Which species and genera are consistently found across wheat-growing regions? 3) How do fungal community compositions vary by region and identification method? 4) What are the dominant lifestyles/strategies of these fungi? By integrating data across global wheat-producing regions, this study aims to identify taxonomic patterns, highlight research gaps, and propose future directions for leveraging phyllosphere fungi in sustainable wheat production.

## 2 Materials and methods

### 2.1 Literature search

Literature published until 2023 identifying fungi in the wheat phyllosphere was mined by combining various search terms (Wheat, *Triticum*, Fungi, Fungal community, Mycobiome, Microbiome, Pathogen, Screening) using Google Scholar and Web of Science. In studies including both fungal and bacterial communities, only the results for the fungal community were extracted. For studies that investigated the mycobiome of various crops, only the results concerning *Triticum* spp. were extracted. Only studies published in English and which identified at least five fungal genera were included.

### 2.2 Data collection

Metadata was extracted from each paper, including identification method (culture-based vs. metabarcoding), sample type (leaf, stem and/or grain), surface sterilization (yes/no), climate zone based on the köppen classification (Köppen, 1936) and geographical location of sampling sites. For a complete list of meta data extracted, see **Supplementary Table 1**. For each study, fungal genera (**Supplementary Table 2**) and species **(Supplementary Table 3**) were extracted and taxonomic names updated using Index Fungorum (https://www.indexfungorum.org/names/names.asp). Higher-level taxonomies were assigned based on the UNITE repository (Abarenkov et al., 2024), and phylogenetic trees were generated using NCBI taxonomy tool (Schoch et al., 2020) and visualized in iTol (Letunic and Bork, 2021). Functional traits, were retrieved from the FungalTraits database (Põlme et al., 2020). Specifically, lifestyle, pathogenicity, endophytic capabilities, growth form and aquatic habitats. Most of the analysis is done on genera level, due to the high number of studies which did not identify fungi to species level.

### 2.3 Assigning wheat pathogenesis

Species found in more than one region and marked as pathogens in FungalTraits were manually checked for records of wheat pathogenesis (**Supplementary Table 4**). Potential to be a wheat pathogen was scored from 0-3, where 0 was for no records of pathogenic interactions with wheat, 1 was for records of rare and mild symptoms, 2 was for rare but severe pathogenesis or common but low pathogenesis, and 3 was for records of large-scale epidemics, high levels of toxins, or major threats to wheat production.

### 2.4 Geographical analysis

Geographic location of all sampling points or, if unavailable, affiliated research institutes were extracted from each study. Studies were grouped into five wheat-growing regions, namely North America, South America, Northern Europe (north of the Pyrenees and the Alps), Mediterranean, and Asia. Regions were assigned based on climatic similarity, wheat production volume (FAO, 1998), and geographic proximity. The countries included in each region are listed in **Supplementary Table 5** and the geographical locations of sampling points are listed in **Supplementary Table 6**. Sampling locations were plotted in QGIS (v. 3.22) with ESRI QuickMap services (QGIS Development Team, 2024) supplemented with global wheat production data (FAO, 1998).

### 2.5 Statistical analysis

For the analysis of spatial autocorrelation, the Mantel test (Sokal, 1979) was used, and a single coordinate was chosen for each paper, which best represented the area of sampling. Calculations were done in R V4.3.2 (R Core Team, 2024). Distances between coordinates were calculated with the distm function in the geosphere (version 1.5-18) package (Hijmans, 2024) 41 using the haversine method. Distances in fungal communities were done using the vegdist function and the final Mantel test was performed using the Mantel function, both from the in the vegan (version 2.6-4) package (Oksanen et al., 2024). A permutational multivariate analysis of variance (PERMANOVA) using the Adonis function in R (vegan version 2.6-4) was performed to assess the influence of sample region, identification method, and pesticide use on fungal community composition.

### 2.6 Ranked abundance analysis

For 32 out of 33 studies where abundance data was available, the top ten most abundant genera were ranked from 1 (most abundant) to 10 (least abundant). Genera not appearing in the top ten were excluded from ranking. The average abundance rank for each genus across all the studies were calculated according to Equation 1:


(1) $$Ab_{ɡenus}=10-\frac{\sum_{i=1}^{n}x_{i}}{n}$$

Where $Ab_{genus}$ is the average ranked abundance for a given genus, $x_{i}$ is the abundance rank of the given genus of the *i*th study and *n* is the total number of studies. Equation 1 inverts the ranked abundance such that one is the most abundant genus and ten is the least abundant genus, a format subsequently used throughout this article. The average rank abundance across regions was calculated by first calculating average ranked abundance for each genus in each region, utilizing the above formula. The ranked abundance for each genus in each region was then converted to average abundance rank across regions according to Equation 2:


(2) $$Ab_{ɡenus,reɡion}=\frac{\sum_{k=1}^{m}Ab_{ɡenus,k}}{m}$$

Where *m* is the number of regions where the genus achieved a ranked score in top 10, $Ab_{genus,k}$ is the average ranked abundance of a genus within the k’ th region and $Ab_{genus,region}$ is the average ranked abundance across regions. The average ranked abundance across regions was then plotted against the number of regions where a given genus was in the 10 most abundant.

## 3 Results

### 3.1 Literature summary

Our literature search identified 33 studies from 26 journals across five major wheat-growing regions (Larran et al., 2007; Perelló et al., 2002; Comby et al., 2016; Hertz et al., 2016; Karlsson et al., 2017; Karlsson et al., 2014; Knorr et al., 2019; Ofek-Lalzar et al., 2016; Rojas et al., 2020a; Rojas et al., 2020b; Sapkota et al., 2015; Vujanovic et al., 2012; Sapkota et al., 2017; Gdanetz et al., 2017; Casini et al., 2019; Schiro et al., 2019; Latz et al., 2021; Wachowska et al., 2013; Al-Khawaldeh et al., 2020; Jiang et al., 2021; Nicolaisen et al., 2014; Huang et al., 2016; Xu et al., 2018; Kaur et al., 2023; Sun et al., 2023; Sun et al., 2020; Zheng et al., 2021; Salamon et al., 2023; Hassanein et al., 2021; Malacrinò et al., 2023; Grudzinska-Sterno et al., 2016; Larran et al., 2002; Blixt et al., 2010) (**Supplementary Table 1**).

Among these, 39% (13 studies) used culture-dependent methods, while 61% (20 studies) used metabarcoding approaches. Approximately 61% of studies (20 studies) employed surface sterilization, aiming to identify endophytic fungi, whereas 39% (13 studies) included both epiphytic and endophytic fungi without sterilization. The studies sampled either single or multiple tissue types; 58% (19 studies) examined wheat heads or grains, 55% (18 studies) focused on leaves and 30% (10 studies) included stems. Wheat crops were grown under different conditions, with most studies sampling wheat grown in fields (20 studies, 61%) or experimental plots (8 studies, 24%). The rest sampled wheat grown in either a greenhouse or a growth chamber (5 studies, 15%).

### 3.2 Geographic distribution of current research

A total of 190 sampling locations across 14 countries and seven different climate zones were identified (**Supplementary Tables 5**, **6**). Using data from Food and Agriculture Organization (FAO), five main regions relevant for global wheat production were assigned to these locations, North America, South America, Northern Europe (north of the Pyrenees and the Alps), Mediterranean and Asia. The distribution of studies was highly unequal between regions and was dominated by Northern Europe (17 studies, 51.5%) and the Mediterranean region (7 studies, 21.2%), followed by Asia (4 studies, 12.1%), South America (3 studies, 9.1%), and North America (2 studies, 6.1%). Notably, no studies were from the region surrounding the Black Sea, including Bulgaria, Romania, Ukraine, Russia, and Turkey, despite this area accounting for nearly 19% of global wheat production (FAO, 1998). Additionally, the number of sampling locations differed between the studies, from one to 51 locations per study.

### 3.3 Fungal diversity in the wheat phyllosphere

**Figure 1:**
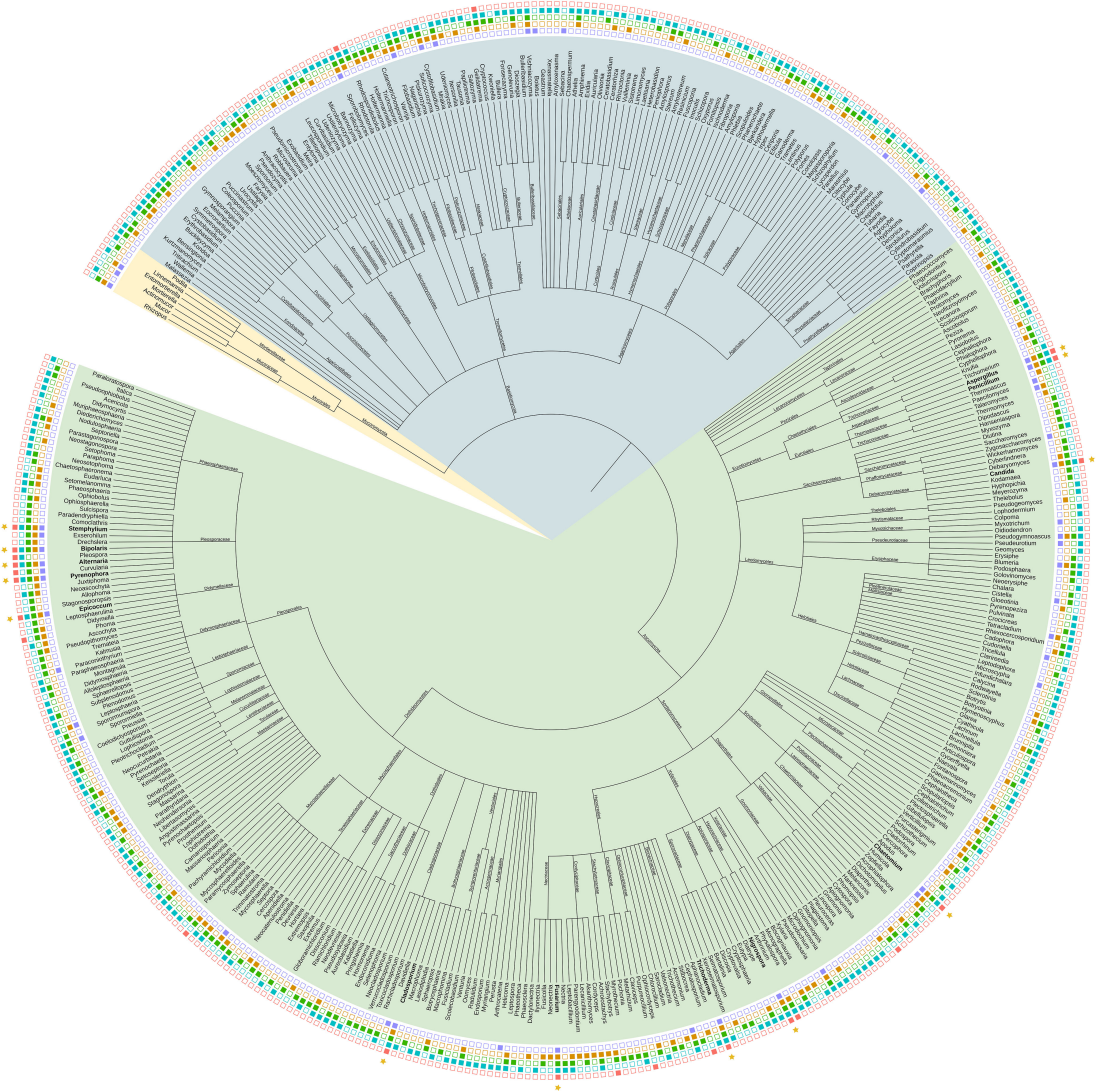
Phylogenetic tree of fungal genera detected in the wheat phyllosphere. Data is extracted from 33 published studies. The tree was constructed using the NCBI taxonomy browser and depicted using iTol. The presence of fungal genera in the identified wheat regions is indicated by color-filled boxes: North America (Blue), South America (Red), Northern Europe (Green), Mediterranean (Orange), and Asia (Purple). Phylum level is shown with colored branches. The green branches represent Ascomycota, blue Basidomycota, and yellow Mucoromycota. Stars indicate genera present in all five regions (High resolution version is available in **Supplementary Figure 1**).

Across all studies, 924 fungal species belonging to 464 genera were identified. The majority belonged to Ascomycota (70.6%), followed by Basidiomycota (27.5%). The phylogenetic relationship of the fungi at genus level is shown in **Figure 1**.

**Figure 2:**
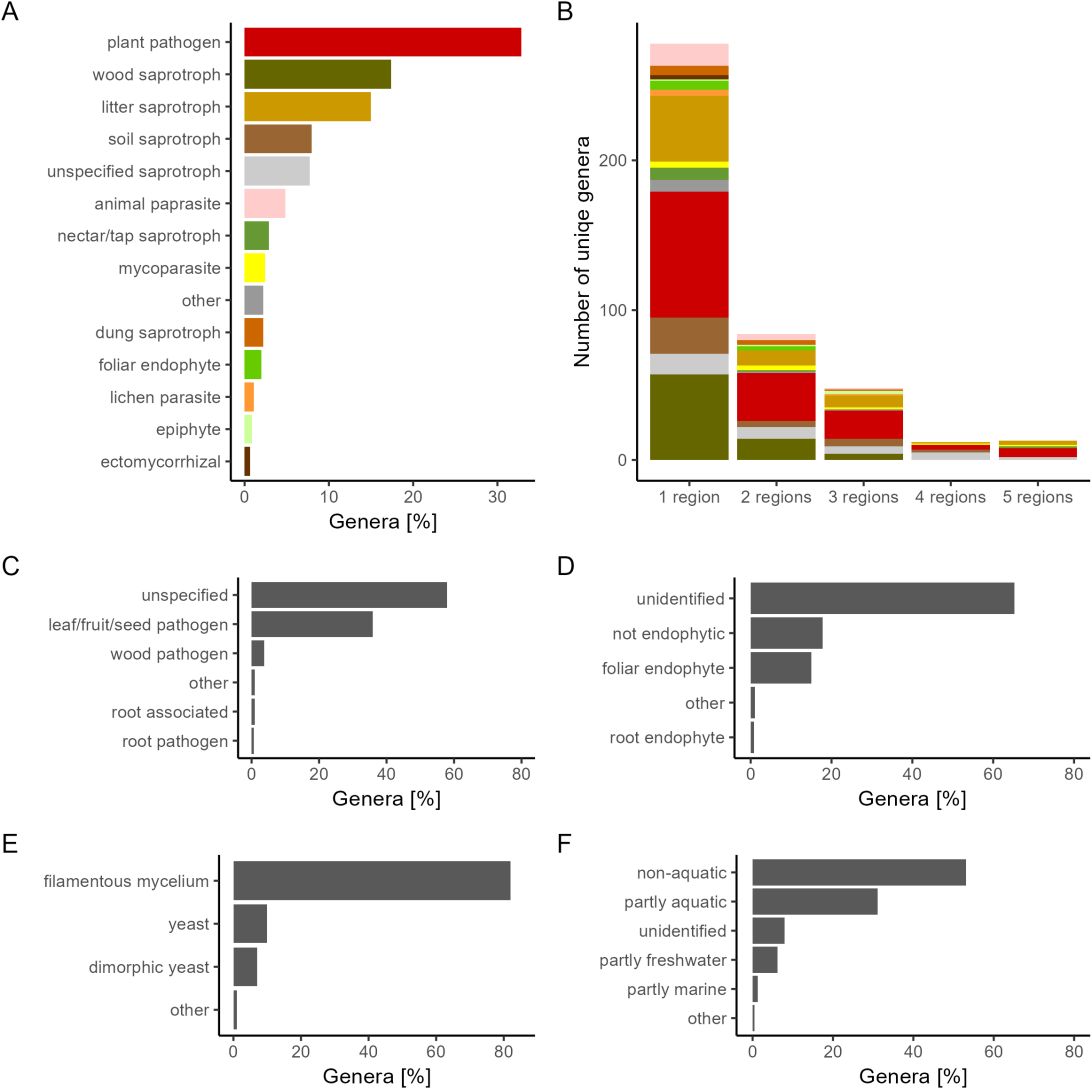
The percentage of genera with a certain trait. Traits were sourced from FungalTraits (Põlme et al., 2020) and summarized for **(A)** primary lifestyle, **(B)** unique genera found across wheat growing regions **(C)** plant pathogenic capacity, **(D)** endophytic interaction capacity, **(E)** growth form, and **(F)** aquatic habitat.

Traits of interest were extracted from the FungalTraits database for each identified genera. A summary is shown in **Figure 2**.

Fungal lifestyles varied greatly. The most common growth forms were filamentous mycelia (81.9%), followed by yeast (9.9%), and dimorphic yeast (7%). Endophytic capacity was undetermined for most genera, but 18% of genera was determined as not endophytic, and 15% of genera was determined to be foliar endophytes. Plant pathogen was the most common primary lifestyle (32.3%) with seeds, fruits, and leaves as the primary targets of infection. Species found in more than one region marked as pathogens in FungalTraits were manually checked for records of wheat pathogenesis (**Supplementary Table 4**). There were 36 genera found in more than one region, marked as plant pathogens as either their primary lifestyle or their secondary lifestyle. Of these, there were 17 genera (47%) in which at least one species was found, with records showing pathogenesis on wheat specifically.

### 3.4 Core wheat phyllosphere mycobiome

No genera were found across all studies or across all countries. However, despite high regional variability, thirteen fungal genera (2.8% of the total genera) were consistently present across all five regions (**Figure 2B**). The majority of the fungal genera (61.9%) were on the other hand found only in a single region. The thirteen fungal genera found in all five regions, henceforth referred to as core genera, were *Cladosporium*, *Epicoccum*, *Alternaria*, *Bipolaris*, *Pyrenophora*, *Stemphylium*, *Aspergillus*, *Penicillium*, *Candida*, *Nigrospora*, *Trichoderma*, *Fusarium*, and *Chaetomium*. At the Species level, *E. nigrum* was the only species found across all five regions. Other frequently recorded species were *Alternaria alternata*, *Alternaria infectoria*, *Chaetomium globosum*, *Cystofilobasidium macerans*, *Pyrenophora tritici-repentis*, *Sporobolomyces roseus*, *Stemphylium vesicarium* and *Vishniacozyma victoriae*, all found across four regions. The taxonomy and traits for the core genera are shown in **Table 1**. A summary of core species are available in **Supplementary Table 7**. All are Ascomycota and all except for the yeast Candida, are filamentous fungi. Most of the genera are partly aquatic and have foliar endophytic capacity. Of the 13 core genera, eight are characterized as commensal or plant growth promoting; *Cladosporium*, *Epicoccum*, *Aspergillus*, *Penicillium*, *Candida*, *Trichoderma*, *Nigrospora* and *Chaetomium*. The remaining five core genera, namely *Alternaria*, *Bipolaris*, *Pyrenophora*, *Stemphylium* and *Fusarium*, are plant pathogens, with species found capable of infecting wheat.

**Table 1:** Core genera in the wheat phyllosphere.

Genus	Rank	Growth form	Lifestyle	Habitats
*Alternaria*	2.4	Filamentous	plant pathogen	indoor, topsoil, human disease, marine, phyllopshere
*Aspergillus*	8.26	Filamentous	unspecified saprotroph	indoor, topsoil, human disease, phyllosphere
*Bipolaris*	8.36	Filamentous	plant pathogen	topsoil, human disease, phyllopshere
*Candida*	9.6	Yeast	nectar/tap saprotroph	human gut, marine, topsoil, human disease
*Chaetomium*	8.9	Filamentous	soil saprotroph	topsoil, phyllopshere
*Cladosporium*	4.75	Filamentous	litter saprotroph	indoor, topsoil, plants, human gut
*Epicoccum*	7.55	Filamentous	litter saprotroph	indoor, topsoil, phyllopshere
*Fusarium*	5.65	Filamentous	plant pathogen	indoor, topsoil, human disease, phyllopshere
*Nigrospora*	9.06	Filamentous	litter saprotroph	topsoil
*Penicillium*	7.52	Filamentous	unspecified saprotroph	indoor, topsoil, human disease, phyllosphere
*Pyrenophora*	9.66	Filamentous	plant pathogen	topsoil
*Stemphylium*	9.23	Filamentous	plant pathogen	topsoil
*Trichoderma*	9.33	Filamentous	Myco-parasite	topsoil, phyllopshere

### 3.5 Abundance patterns

**Figure 3:**
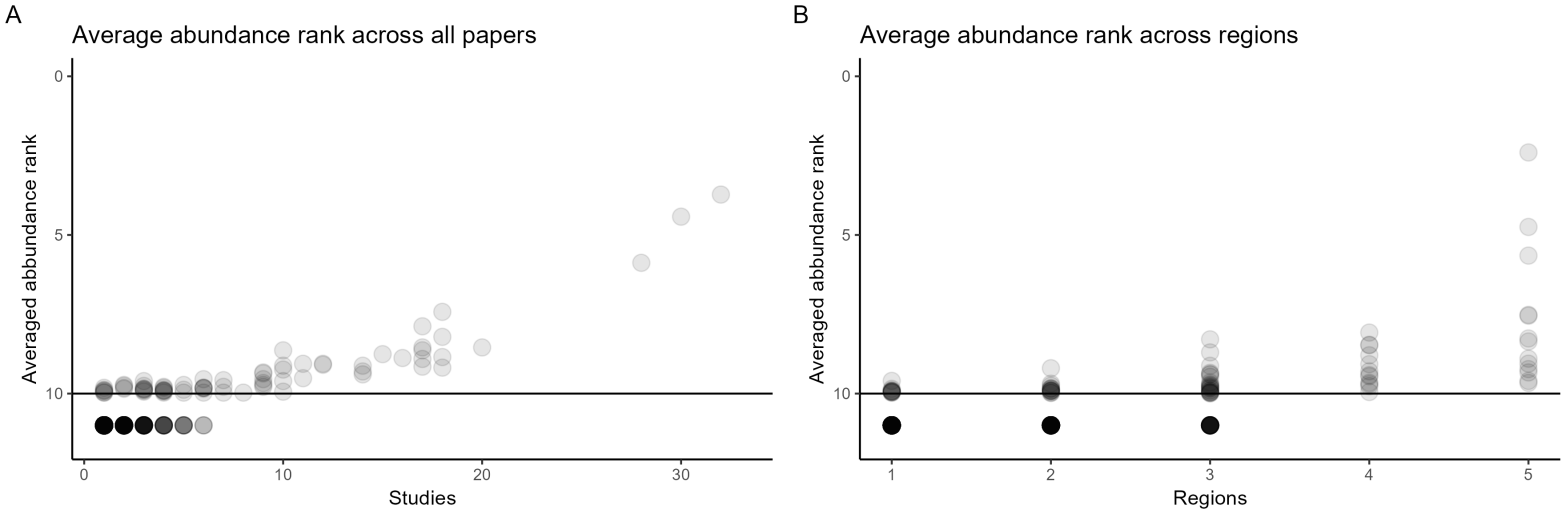
Scatter plot of the average rank of genera as a function of how many times each genus was recorded across studies **(A)** and regions **(B)**. In each study, the top 10 genera were ranked 1-10, with one being the most abundant. Genera not recorded as the top 10 most abundant genera in any paper are plotted below the line. The intensity of the dots (alpha = 0.2) reflects the number of genera of a particular abundance rank.

To determine if genera found across multiple studies and regions were also the most abundant in individual studies, the top ten most abundant genera were ranked for each study. A table for average ranked abundance for each genus can be found in **Supplementary Table 8**. The average across studies and regions are plotted in **Figure 3**.

Across both studies and regions, the most commonly found genera were also the highest ranked genera (with 1 being the highest rank). All the genera found in four or more regions had across studies a ranked abundance within the top 10. The top three most abundant and widespread genera were *Alternaria*, *Cladosporium*, and *Fusarium*, which also consistently were the highest ranked across studies.

### 3.6 Geographic variation in mycobiome composition

**Figure 4:**
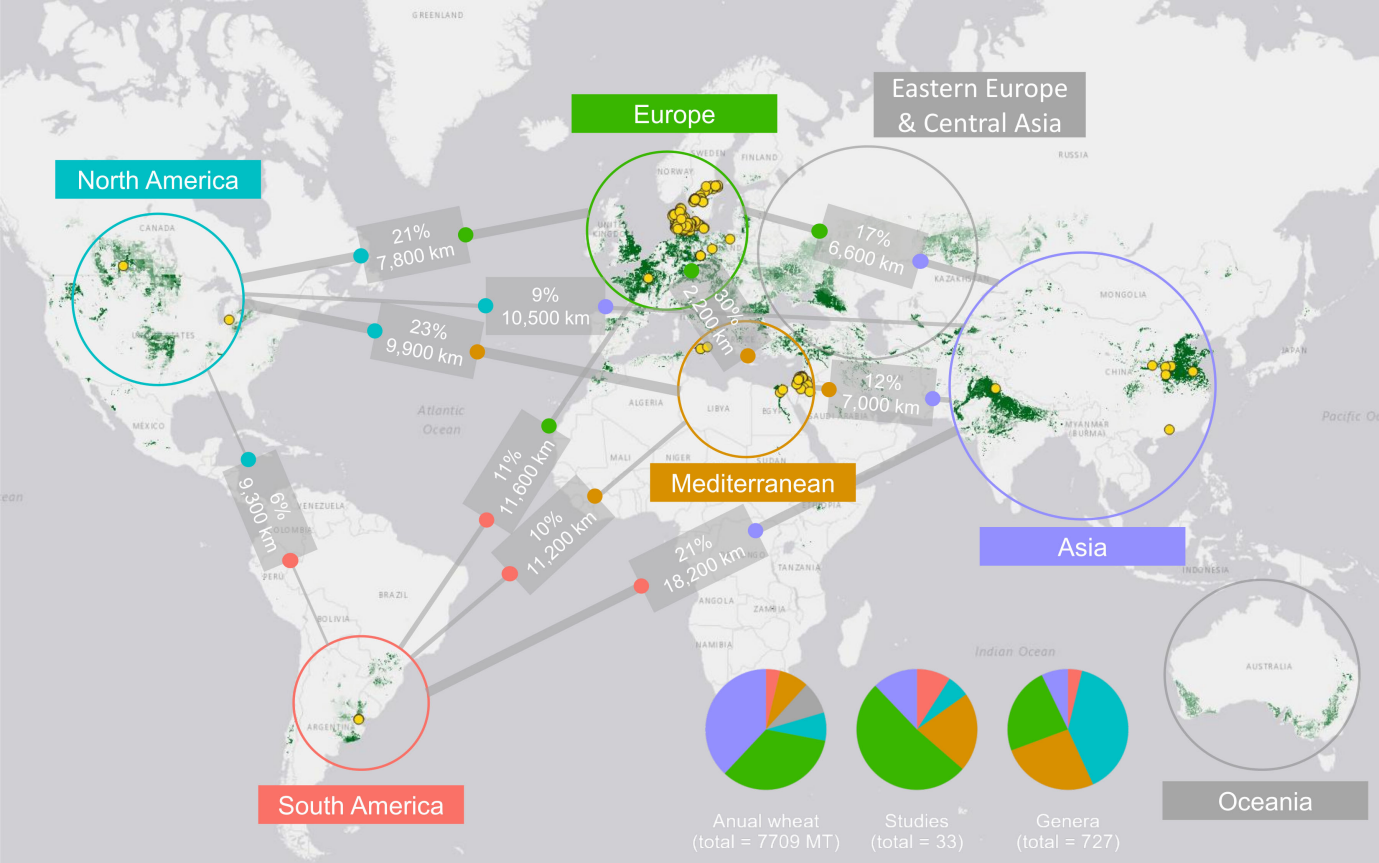
A world map showcasing wheat production, sample locations, and the percentage of shared genera between regions. The map was made using QGIS and data from FAOSTAT. The areas of wheat production are depicted in dark green, while the yellow dots on the map represent the sampling points within each wheat-growing region. Lines connecting each region indicate the percentage of shared genera and the distance between them. Pie charts show the distribution of annual wheat production, the number of studies, and the number of genera across regions.

Similarities between regions were determined by comparing the number of genera shared between each combination of regions (**Figure 4**). Regions shared an average of 26.7 genera (SD = 31.5). The highest similarity was observed between North America and the Mediterranean (95 shared genera), while the lowest similarity occurred between South America and Asia (13 shared genera). Similarities within each region, defined here as the number of genera found more than once, were on average 27.5 genera (SD = 32.2), which represents 13.6% (SD = 17.7).

**Figure 5:**
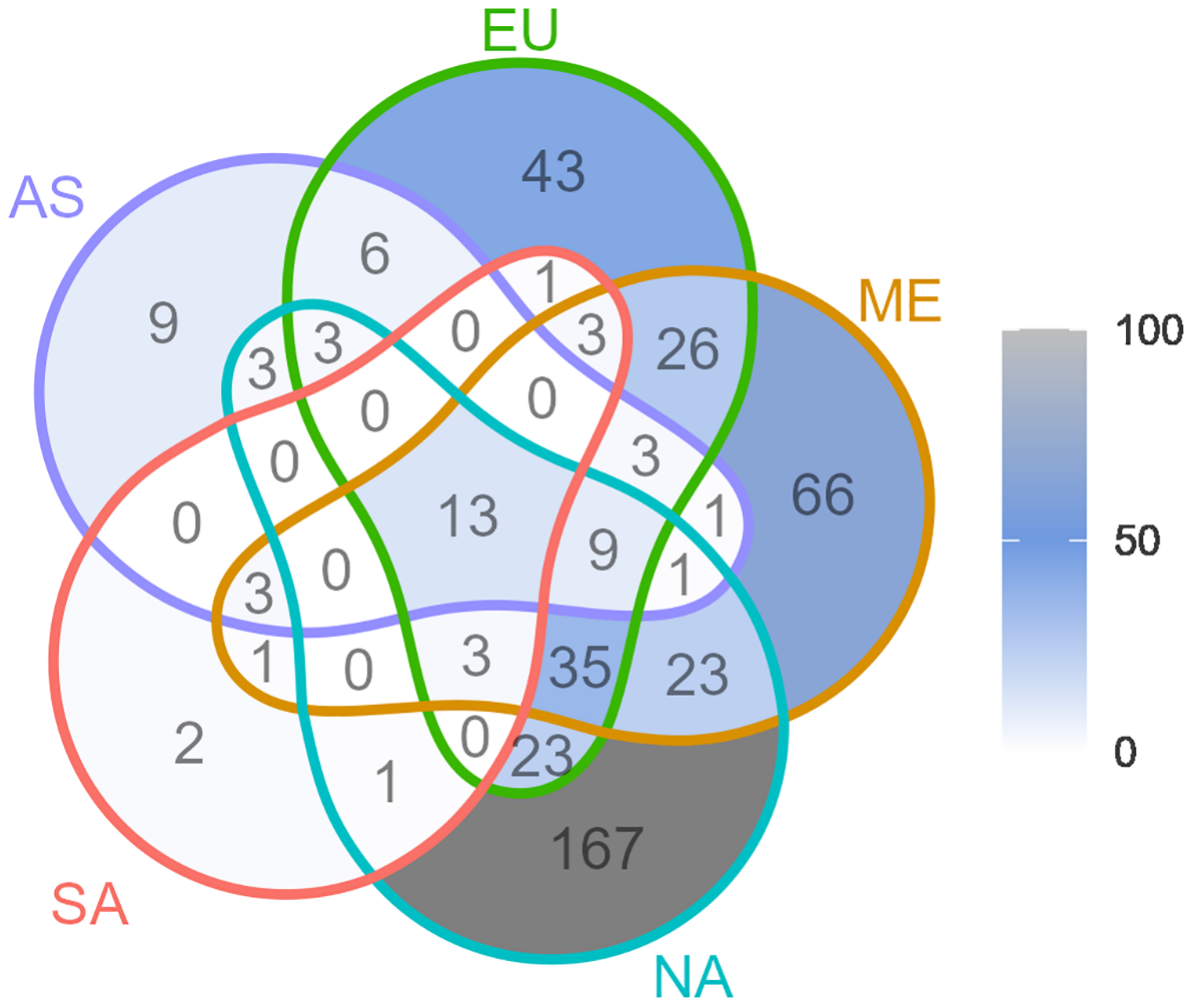
Venn diagram of genera shared between the wheat growing regions North America (NA), South America (SA), Northern Europe (EU), Mediterranean (ME), and Asia (AS). Numbers indicate shared genera within a specific intersection. See **Figure 4** for the exact locations of studies and regions.

The Venn diagram in **Figure 5**, visualizes the distribution of shared genera between regions. The region with the largest number of genera not shared with any other regions was North America. This was followed by the Mediterranean region. The region where most genera was shared (but also with the fewest genera in total) was South America.

### 3.7 Factors influencing fungal community composition

An Adonis test was performed to identify which potential factors significantly affected the community composition, namely identification method, wheat growing region, pesticide use, surface sterilization, and climate zone (**Table 2**). As the Adonis test is sensitive to dispersion, a dispersion pre-test was also performed. Three factors were not significantly dispersed: identification method, use of pesticides, and whether the plant sample was surface sterilized or not. Of these three factors, the identification method and if leaves were surface sterilized or not significantly affected fungal community composition at genus level (**Table 2**).

**Table 2:** Adonis and dispersion output of analyzed factors potentially affecting the fungal phyllosphere community composition.

Factor	Adonis p	Dispersion p
Identification method^1^	0.000999 ***^3^	0.93
Wheat growing region^2^	0.000999 ***	0.031 *
Pesticides	0.07193	0.105
Surface sterilization	0.001998 **	0.06.
Climate	0.000999 ***	0.001 ***
Country	0.000999 ***	0.001 ***

^1^Cultured and identified versus meta-barcoding. ^2^See **Figure 4**. ^3^ Significance codes: 0 ‘***’, < 0.001 ‘**’, < 0.01 ‘*’, < 0.05 ‘.’, < 0.1 ‘ ’ 1.

**Figure 6:**
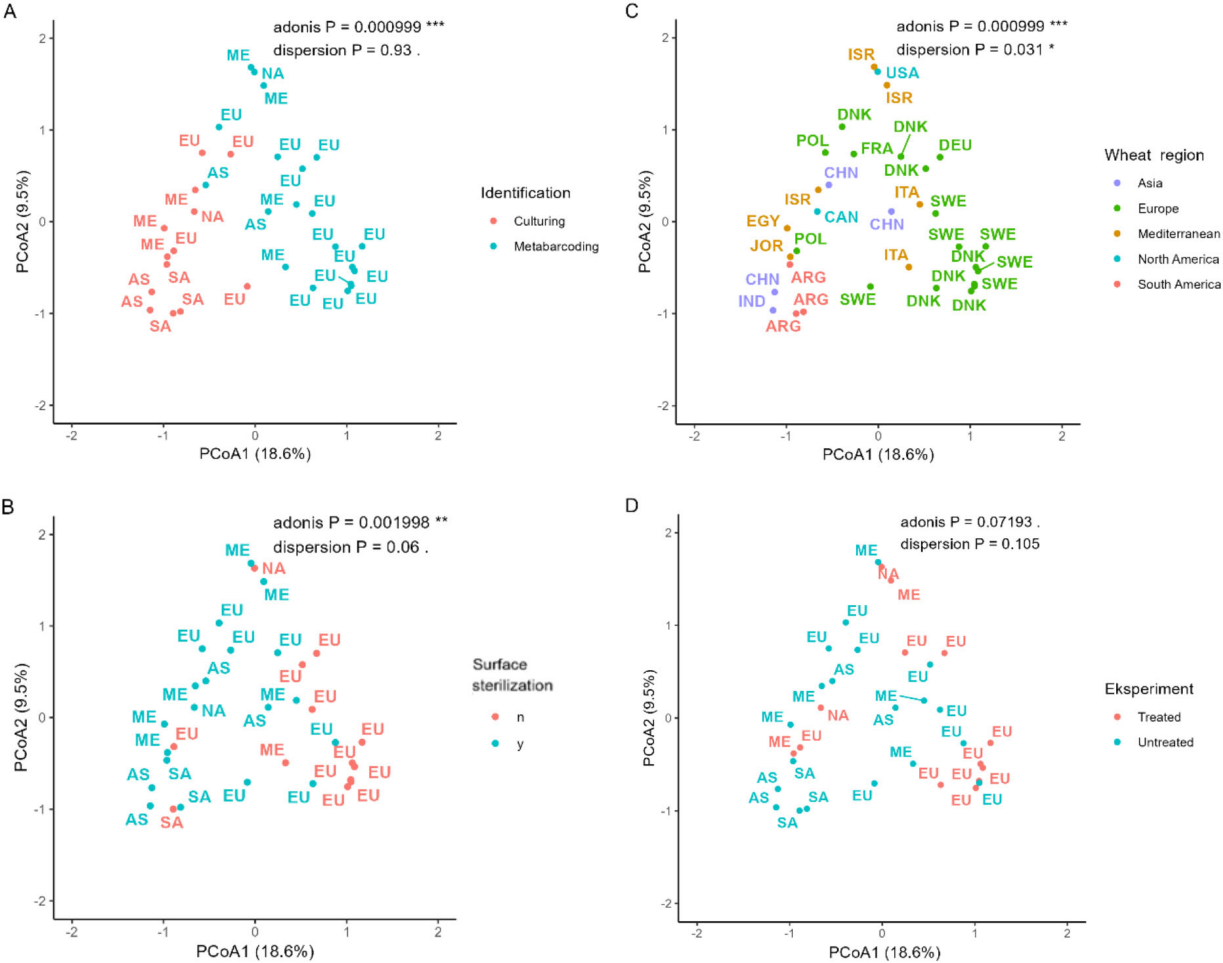
PCoA plot based on Jaccard dissimilarities. Each point is a study, and the overlaid colors represent identification methods **(A)**, surface sterilization **(B)**, wheat growing region **(C)**, or use of antifungal compounds **(D)**. Wheat region abbreviations: NA, North America; SA, South America; EU, Northern Europe; ME, Mediterranean; AS, Asia. Country abbreviations: ARG, Argentina; CAN, Canada; CHN, China; DNK, Denmark; EGY, Egypt; FRA, France; DEU, Germany; IND, India; ISR, Israel; ITA, Italy; JOR, Jordan; POL, Poland; SWE, Sweden; USA, USA.

Distances in community composition between studies were calculated using the Jaccard method for the presence/absence data, at genus level and depicted in a principal coordinate analysis (PCoA) plot (**Figure 6**). The plot is depicted four times each overlayed with either wheat regions, identification methods, surface sterilization and pesticide use to visualize patterns. The first principal coordinate explained 18.6% and the second principal coordinate explained 9.5% of the variation, in total explaining 28.1% of the variation in the community composition. The clearest separation of data points was seen when the identification method was overlayed (**Figure 6A**). Interestingly, only Northern Europe clustered separately from the other regions (**Figure 6**).

**Figure 7:**
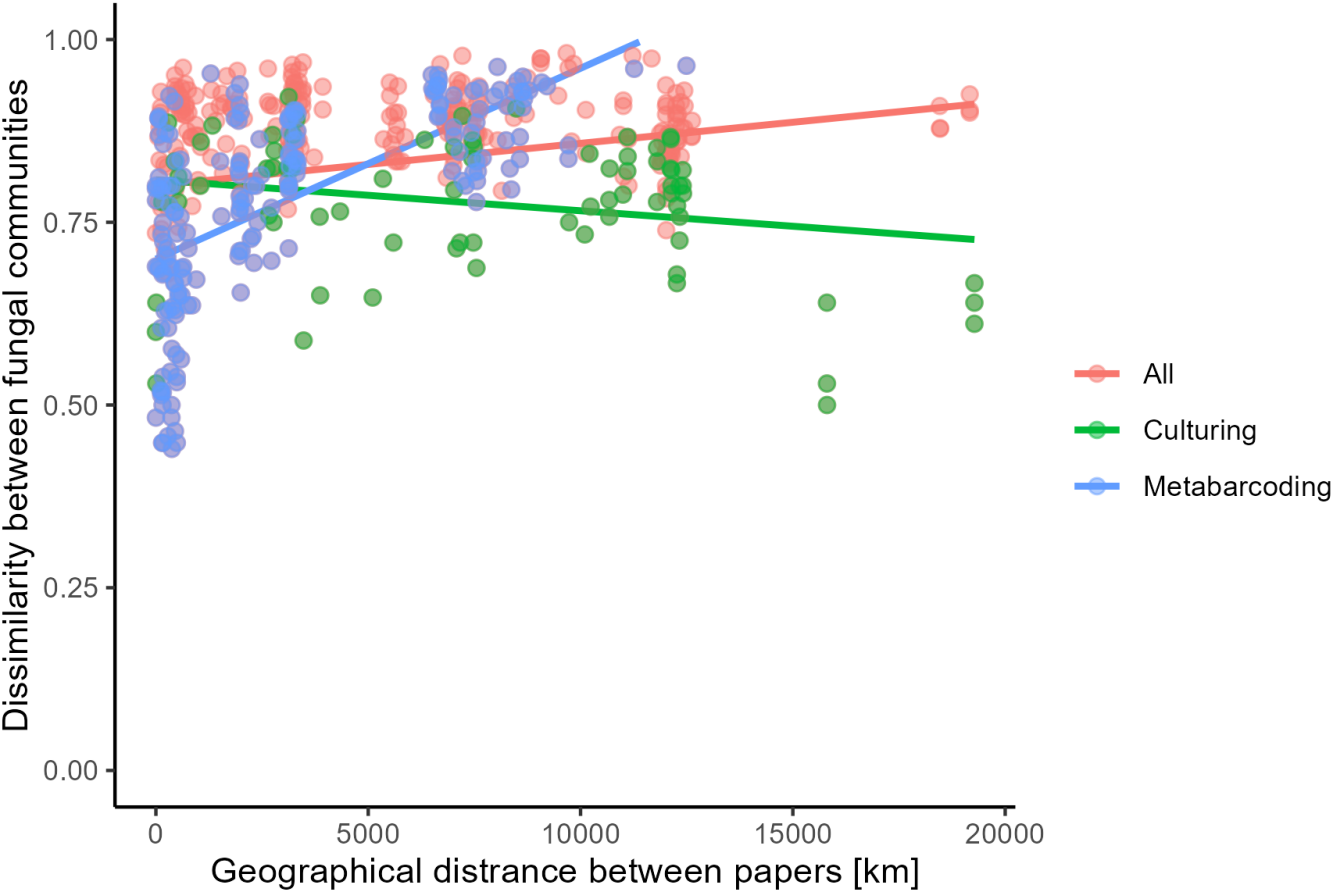
Distance in fungal community (Bray Curtis dissimilarity) over geographical distance (km) between all studies (red), and studies identifying fungi using culturing (green) or metabarcoding (blue).

To determine whether increased distance between sampling sites is associated with an increased dissimilarity between fungal communities, a Mantel test was performed. Given that the method of identification significantly influenced community composition (**Table 2**, **Figure 6A**), three Mantel tests were performed. One on all studies and two where studies were grouped based on identification method. Results from the Mantel tests are shown in **Table 3** and visualized in **Figure 7**. Both for all studies together and for metabarcoding studies there was a significant positive correlation between community dissimilarity and geographical distance. This pattern was not observed for culture-based studies.

**Table 3:** Mantel statistics.

Grouping	r	Significance
All	0.2889	0.0018 **
Culturing	-0.214	0.9408
Metabarcoding	0.6562	0.0001 ***

Significance codes: 0 ‘***’, < 0.001 ‘**’, < 0.01 ‘*’, < 0.05 ‘.’, < 0.1 ‘ ’ 1.

## 4 Discussion

The need for sustainable alternatives to fungicide has led to an increased interest in the plant microbiome in general and specifically for the discovery of microbes capable of promoting or protecting plant growth. Multiple promising candidates have been identified (Khan et al., 2007; Sarrocco et al., 2019; Rojas et al., 2020b), some of which are already on the market (Rush et al., 2021). However, the many promising candidates and the relatively few products underline the major challenge of transitioning from the simple and controlled laboratory and greenhouse settings to the unpredictable and complex field conditions (Parnell et al., 2016).

### 4.1 Key findings

The present study documented the impressive fungal diversity associated with the phyllosphere of a single domesticated crop, wheat, in which over 300 genera were identified. As many studies did not identify the fungi to species level, the number of species to which this corresponds will therefore only be a guess, but a conservative estimate would certainly be above the 924 species that was recorded. A core wheat mycobiome comprising of 13 genera was identified. The available studies highlight how skewed sampling is between regions. Neither genera nor species richness were saturated when only the currently sampled regions were included (**Supplementary Figure 2**). We expect the number of genera and species identified in the wheat phyllosphere to increase, and to approach saturation, following more in-depth sampling across the entire wheat growing area. These findings and more will be discussed further in the following sections.

### 4.2 Non-sampled wheat regions

The low similarities between fungal phyllosphere communities, both within and between regions, stresses the need for more sampling. Even though the data was extracted from five continents, it was largely dominated by samples from Northern Europe and the Mediterranean (representing 72% of total studies). This bias is most likely the result of active wheat microbiome research groups, e.g. at universities in Denmark, Sweden, and Israel. Many important wheat-producing areas, including China (134 million tons), India (104 million tons), Russia (75 million tons), and the United States (53 million tons), which account for 48% of global annual wheat production (FAO, 2019), are underrepresented, as they contribute to only 12% of the total studies analyzed in this meta-analysis. Coordinated global sampling efforts, such as previously done by Tedersoo et al. or Dunn et al. for other habitats, could represent a major step forward toward comprehensive identification of the global wheat mycobiome (Tedersoo et al., 2014; Dunn et al., 2013).

### 4.3 It is common to be rare

From the regions studied to date, we could not identify any species or genera that were identified across all 33 studies. This, combined with the high number of genera found in only a single study and the low number of genera shared within and between regions, indicates that the wheat phyllosphere is a complex mycobiome inhabited by many rare fungi. Agricultural wheat is an annual plant. Each year present a new chance for fungi to colonize the plant, which could explain why it is common to be rare. The low sampling intensity in certain regions is likely to also contribute to the low similarity between regions.

### 4.4 A core of 13 genera across regions were found

Of the most common genera, 13 were found across all five regions: *Cladosporium*, *Epicoccum*, *Alternaria*, *Bipolaris*, *Pyrenophora*, *Stemphylium*, *Aspergillus*, *Penicillium*, *Candida*, *Nigrospora*, *Trichoderma*, *Fusarium*, and *Chaetomium*. Of these, five genera, *Alternaria*, *Cladosporium*, *Fusarium*, *Penicillium*, and *Epicoccum*, were also the most abundant across regions. Although these 13 genera are defined as core wheat phyllosphere mycobiomes, they are not unique to wheat. Most are common genera found in both the phyllosphere and rhizosphere of other cereals (Sapkota et al., 2015), plants in general (Sohrabi et al., 2023), soil (Tedersoo et al., 2014) and even in humans (Köhler et al., 2015) (**Table 1**). Of the 13 core genera, seven comprised of commensal or plant growth-promoting species, while six were wheat specific pathogens. More studies comparing fungal community composition across a variety of hosts, such as other crops and nearby vegetation within each region, are needed to identify if any species are unique to wheat, and the extent to which fungal phyllosphere communities represent the selective pressure of the host versus the meta community of the region. Including relevant species of these 13 genera in multi-species synthetic communities (SynCom), mimicking global wheat phyllosphere communities could be beneficial for future biocontrol success. We suggest that for any potential biological control agent to effectively integrate into native wheat microbiome, it would need to be able to co-exist, show positive or at least neutral interactions with these genera.

### 4.5 Sampling method rules above geographical areas

Across all studies, regardless of the sampling region, the PCoA plots (**Figure 6A**) and the Adonis test (**Table 2**) showed a clear separation of fungal communities identified with either barcoding or culturing methods. This indicates that the fungal identification method had a more significant impact on the identified community composition than geographical distance. However, when metabarcoding data was analyzed alone, with a Mantel test, a clear signal was observed by a decrease in community similarity with increased geographical distance. We found that most of the metabarcoding studies were also from the most intensively sampled region (Europe) and that these studies clustered together in the ordination space (**Figure 6C**). Possibly, the studies using culturing clustered separately together because this method favors the most common and cultivable fungi, thereby finding fewer rare genera unique to the region.

This highlights the need for more deep sequencing of all wheat growing regions, to find differences in the fungal microbiome that is not caused by our method of sampling. Different sampling and identification methods can affect observed fungal diversity by the different biases/selective opportunities that each method has. Metabarcoding is a high throughput tool which will find more rare fungi. In this study, more fungal genera were identified in studies using metabarcoding (average of 47 genera per study) than culturing (average of 20 genera per study). Whereas metabarcoding approaches are a promising tool in accurately describing complete wheat phyllosphere fungal communities, their results may be biased by primer selectivity and sequencing errors, which if not corrected can artificially inflate/disregard the species count of specific groups (Zou et al., 2019). By contrast, culturing favors fast-growing fungi and is more labor-intensive, thereby often finding fewer unique genera but allows for better identification and characterization of the fungi once in culture. It should be noted that recent improvements in high-throughput cultivation and sequencing could in future studies close the gap between culturing and metabarcoding species counts (Collado et al., 2007; Li et al., 2023). Importantly, this indicates that regions might in fact share more genera than reported in this study, which were not recognized due to different identification methods. We expect that if all regions were equally sequenced using a standardized metabarcoding approach, the number of shared genera would increase and the number of genera recorded could saturate.

### 4.6 The use of pesticides had little effect

Counterintuitively, we saw only a small effect of pesticides treatment on the fungal community composition. Similar marginal effects of pesticides use have been documented in literature before. Both (Karlsson et al., 2014; Knorr et al., 2019) did find an effect on the abundance of certain species but only a moderate effect on species composition, all which was affected by fungicide choice, timing and dose. In our study, it was not possible to identify the effect of other factors such as the surrounding pool of fungi, climate, plant organ, plant developmental stage, wheat cultivar, or genotype, either because they were not recorded or because there were too few studies representing each factor. More focus on the effect of these factors is needed in future studies.

### 4.7 Yeast has a high abundance but low diversity

We found relatively fewer yeast genera (16.7%) than filamentous fungal genera (80.7%). Since the filamentous growth form has the potential to grow internally between the leafs cells, taking up nutrients from within the host, this might allow a higher taxonomic diversity. In contrast, epiphytically growing yeast has to specialize in adapting to the leaves harsh surface environment. We tested whether the use of surface sterilization in 13 out of 33 studies could have affected the observed diversity. However, interestingly, studies using surface sterilization found more yeasts or yeast-like growth forms compared to studies not using surface sterilization, even though they observed approximately the same total number of genera (**Supplementary Table 9**). This suggests that either surface sterilization methods are not efficient enough to remove epiphytically growing fungi or that more yeast grow endophytically than expected. The relatively fewer yeast genera found did not indicate that yeast is not abundant in the wheat phyllosphere. We could see that seven out of the top twenty genera with the highest average ranked abundance across studies were yeasts (*Sporobolomyces*, *Cryptococcus*, *Dioszegia*, *Filobasidium*, *Udeniomyces*, *Rhodotorula*, and *Vishniacozyma*). The data therefore indicates that yeast has a low diversity compared to filamentous but are equally abundant.

### 4.8 Few endophytes or over representations of pathogens?

Using the FungalTraits database to assign lifestyle strategies to genera, we most commonly found plant pathogens as the lifestyle strategy. This was surprising, considering that most studies reported that fungi were isolated from symptom-free samples. However, our result was heavily skewed by genera that were found only once. When looking at genera found in more than one region, most were not characterized as plant pathogens, and less than half of them were known to infect wheat. Clearly, a trait as plant pathogen, especially a pathogen on above ground wheat structures, is much narrower than what predicted by FungalTraits. Not all species within a genus may be pathogens, and not all pathogens within a genus may infect wheat. From other fungal pathogens it is known that even small genetic differences may determine if these are pathogenic or not (Skovgaard et al., 2002; Stukenbrock and Mcdonald, 2007).

### 4.9 Knowledge gaps and suggestions for future studies

Our approach allowed us to integrate relevant studies using different species recognition methodologies into one analysis. By doing so, we emphasized the complexity of the wheat phyllosphere mycobiome and identify a core of 13 wheat phyllosphere fungi across five major wheat-growing regions. However, we should acknowledge that our method is limited by the studies that were available to us and our reliance on species or genera originally proposed by the authors of the studies. Although we updated the taxonomy to the most recently accepted one, there is still a chance that we have included misidentifications or that the authors across time and locations have worked with different frameworks for species designation. However, our approach allowed us to identify the following knowledge gaps. 1) Many important wheat-producing areas, including China, India, Russia, and the United States, are underrepresented in wheat phyllosphere mycobiome studies, despite contributing significantly to global wheat production. 2) It remains unclear how different environmental and agricultural factors influence the composition of wheat-associated fungal communities. 3) More studies are needed to determine whether certain fungal species or genera are unique to wheat. 4) Yeasts are abundant but exhibit low diversity in the wheat phyllosphere. The extent of their ecological roles and interactions with other fungi requires further investigation. 5) Many fungi identified in wheat phyllosphere studies are classified as potential pathogens, but their actual roles (pathogenic, mutualistic, or commensal) remain uncertain. 6) Different studies use varying methods for identifying fungi, leading to inconsistent results.

A standardized approach is needed to allow better comparisons across studies and regions. Future studies of the wheat phyllosphere mycobiomes will hopefully increase the knowledge of the under-sampled regions and allow for a comprehensive understanding of the wheat phyllosphere without methodological biases. Ultimately, the obtained knowledge may contribute to the development of wheat protection and growth enhancement methods alternative to the extensive use of pesticides and fertilizers.

## Data Availability

The original contributions presented in the study are included in the article/**Supplementary Material**. Further inquiries can be directed to the corresponding author.
