# Supplementary figures and images for: Taxonomic diversity in the global wheat phyllosphere mycobiome – a meta analysis
Source: Front Plant Sci. 2025 Jul 30;16:1597807. doi: 10.3389/fpls.2025.1597807 (PMC12343630; doi:10.3389/fpls.2025.1597807)

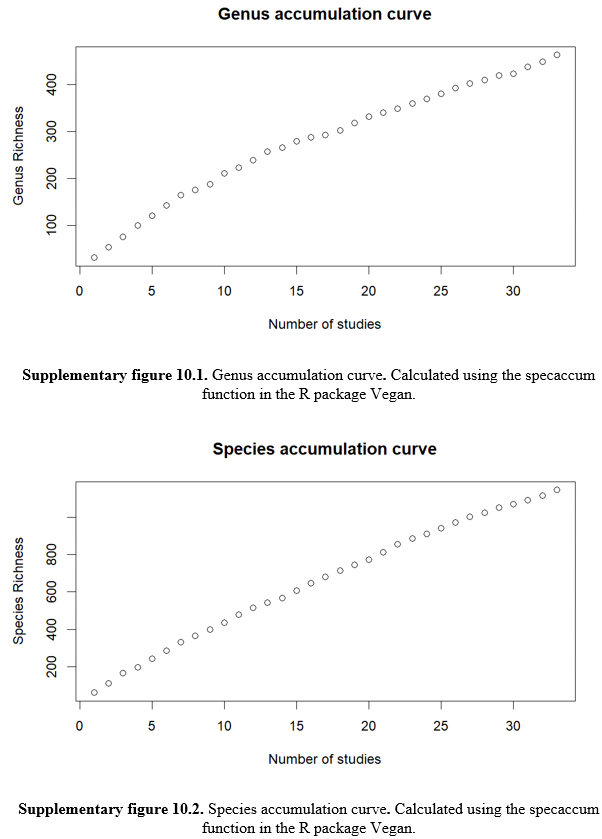

Supplement: Supplementary file 11 [file Image2.png]
